# Harnessing Expression Data to Identify Novel Candidate Genes in Polycystic Ovary Syndrome

**DOI:** 10.1371/journal.pone.0020120

**Published:** 2011-05-17

**Authors:** Michelle R. Jones, Angela Chua, Yii-Der I. Chen, Xiaohui Li, Ronald M. Krauss, Jerome I. Rotter, Richard S. Legro, Ricardo Azziz, Mark O. Goodarzi

**Affiliations:** 1 Division of Endocrinology, Diabetes and Metabolism, Department of Medicine, Cedars-Sinai Medical Center, Los Angeles, California, United States of America; 2 Department of Obstetrics and Gynecology, Cedars-Sinai Medical Center, Los Angeles, California, United States of America; 3 Medical Genetics Institute, Cedars-Sinai Medical Center, Los Angeles, California, United States of America; 4 Department of Medicine, David Geffen School of Medicine, University of California Los Angeles, Los Angeles, California, United States of America; 5 Department of Obstetrics and Gynecology, David Geffen School of Medicine, University of California Los Angeles, Los Angeles, California, United States of America; 6 Children's Hospital Oakland Research Institute, Oakland, California, United States of America; 7 Department of Obstetrics and Gynecology, Pennsylvania State College of Medicine, Hershey, Pennsylvania, United States of America; Rutgers University, United States of America

## Abstract

Novel pathways in polycystic ovary syndrome (PCOS) are being identified in gene expression studies in PCOS tissues; such pathways may contain key genes in disease etiology. Previous expression studies identified both dickkopf homolog 1 (*DKK1*) and DnaJ (Hsp40) homolog, subfamily B, member 1 (*DNAJB1*) as differentially expressed in PCOS tissue, implicating them as candidates for PCOS susceptibility. To test this, we genotyped a discovery cohort of 335 PCOS cases and 198 healthy controls for three *DKK1* single nucleotide polymorphisms (SNPs) and four *DNAJB1* SNPs and a replication cohort of 396 PCOS cases and 306 healthy controls for 1 *DKK1* SNP and 1 *DNAJB1* SNP. SNPs and haplotypes were determined and tested for association with PCOS and component phenotypes. We found that no single nucleotide polymorphisms were associated with PCOS risk; however, the major allele of rs1569198 from *DKK1* was associated with increased total testosterone (discovery cohort P = 0.0035) and dehydroepiandrosterone sulfate (replication cohort P = 0.05). Minor allele carriers at rs3962158 from *DNAJB1* had increased fasting insulin (discovery cohort P = 0.003), increased HOMA-IR (discovery cohort P = 0.006; replication cohort P = 0.036), and increased HOMA-%B (discovery cohort P = 0.004). Carriers of haplotype 2 at *DNAJB1* also had increased fasting insulin, HOMA-IR, and HOMA-%B. These findings suggest that genetic variation in *DKK1* and *DNAJB1* may have a role in the hyperandrogenic and metabolic dysfunction of PCOS, respectively. Our results also demonstrate the utility of gene expression data as a source of novel candidate genes in PCOS, a complex and still incompletely defined disease, for which alternative methods of gene identification are needed.

## Introduction

Familial aggregation and twin studies have established a genetic etiology for polycystic ovary syndrome (PCOS) [1]. The hallmark of PCOS is hyperandrogenemia; however, insulin resistance, pancreatic beta cell dysfunction and chronic inflammation are frequently present [1], [2], [3]. Many previous candidate gene studies focused on genes from androgen synthesis and insulin signaling pathways. Few susceptibility genes are widely agreed upon, potentially because candidate gene selection has been based incomplete understanding of the disorder.

Recently a number of expression studies (mRNA and protein) have been performed in PCOS tissues, including ovary [4], omental fat [5] and lymphocytes [6]. Remarkably few genes have been reported as differentially expressed in PCOS tissues in more than one study. One such gene is dickkopf homolog 1 (*DKK1*), an inhibitor of the Wnt signaling pathway and cell growth repressor [4], [5]. *DKK1* interacts with the Wnt coreceptor low-density lipoprotein receptor-related proteins 5 and 6 and influences several functions, including embryogenesis and cell cycle regulation in cancer pathways [7]. It has been reported as being under expressed in omental fat [5] and over expressed in cultured ovarian theca [4] from PCOS subjects.

The second gene we selected for analysis in this study is DnaJ (Hsp40) homolog, subfamily B, member 1 (*DNAJB1*), whose expression was decreased in ovaries from PCOS women [8]. *DNAJB1* was also selected as a positional and functional candidate. It acts in concert with molecular chaperones to regulate protein folding, protein complex assembly and disassembly, and transport of proteins across cellular membranes, particularly in the androgen signaling pathway, and is under transcriptional regulation by insulin [9]. It is also located within the chromosome 19p13.2 linkage region that has been identified in PCOS susceptibility [10], implicating *DNAJB1* as a potential positional candidate.

Because logical candidates have led to few successes in PCOS genetics, novel methods of candidate gene selection are needed. In the present study we used published expression data to identify putative molecular targets; selecting two genes, *DKK1* and *DNABJ1*, from such analysis for association study with PCOS, conducted in two independent case/control cohorts. A SNP in *DNABJ1* was associated with a measure of insulin resistance in women with PCOS in both cohorts.

## Materials and Methods

### Ethics statement

All subjects gave written informed consent; each study was approved by the Institutional Review Boards of the recruiting centers and Cedars-Sinai Medical Center.

### Subjects and phenotyping

#### Discovery Cohort

We studied 335 unrelated White PCOS patients and 198 White control women recruited at two centers, the University of Alabama at Birmingham (UAB; 287 PCOS and 187 controls) and Cedars-Sinai Medical Center (CSMC; 48 PCOS and 11 controls). Cases were premenopausal, non-pregnant, on no hormonal therapy, including oral contraceptives, for at least three months; all PCOS subjects met 1990 NIH criteria [11]. Parameters for defining hirsutism, hyperandrogenemia, ovulatory dysfunction, and exclusion of related disorders were previously reported [12]. Controls were healthy women, with regular menstrual cycles and no evidence of hirsutism, acne, alopecia, or endocrine dysfunction and had not taken hormonal therapy (including oral contraceptives) for at least three months. Controls were recruited by word of mouth and advertisements calling for “healthy women.”

#### Replication Cohort

We assembled a cohort of 396 unrelated White PCOS patients and 306 White control women. The replication cohort was constituted from three sources: 380 PCOS subjects (all meeting 1990 NIH criteria) and 71 healthy controls previously recruited by R. S. Legro [13], 16 PCOS subjects and 2 healthy controls recruited at Cedars-Sinai Medical Center using the same criteria as those used in the discovery cohort; and 233 white control women derived from the Cholesterol and Atherosclerosis Pharmacogenetics (CAP) study, a component of the Pharmacogenomics and Risk of Cardiovascular Disease (PARC) Study [14].

Subjects recruited at UAB and CSMC were evaluated per a previously described protocol [12]. Fasting glucose and insulin were also obtained in a subset (70%) of the cases (non-diabetic). The subset of subjects with fasting glucose and insulin did not differ demographically or hormonally from the study subjects overall. The computer-based homeostasis model assessment (HOMA, www.dtu.ox.ac.uk/homa) was used to calculate indices of insulin resistance (HOMA-IR) and insulin secretion (i.e. percent beta-cell function or HOMA-%B) utilizing the fasting glucose and insulin levels. This computer model was also applied to generate HOMA data in the replication cohort, on whom fasting insulin and glucose were measured in >90% of subjects. Table 1 presents clinical characteristics of both cohorts.

**Table 1:** Clinical Characteristics of PCOS and control subjects.

	Discovery		Replication	
	Control(n = 198)	PCOS(n = 335)	Control(n = 306)	PCOS(n = 396)
Age (yr)	32.5 (17.0)	27.0 (11.1)*	51.0 (23.7)	28.0 (9.0)*
BMI (kg/m^2^)	24.7 (5.9)	33.4 (14.7)*	25.9 (8.3)	34.9 (12.3)*
Total testosterone (nmol/l)	1.40 (0.94)	2.63 (1.14)*	1.0 (0.52)	2.43 (1.11)*
DHEAS (µmol/l)	2.58 (2.04)	5.68 (4.57)*	3.70 (1.75)	5.72 (3.94)*
Insulin (pmol/l)	41.4 (39.0)	96.0 (108.0)*	72.0 (45.0)	126.0 (96.0)*
Glucose (mmol/l)	4.77 (0.56)	4.77 (0.72)	5.01 (0.68)	4.86 (0.64)
HOMA-IR	0.95 (0.84)	2.01 (2.13)*	1.58 (0.96)	2.69 (1.83)*
HOMA-%B	104.1 (57.0)	166.9 (108.8)*	129.5 (58.3)	196.6 (81.0)*

*P<0.001 compared to control group. Data are median (interquartile range). In the replication cohort, androgen measurements were available only in the cases and controls recruited by R. S. Legro.

Abbreviations: BMI: body mass index; mFG: modified Ferriman-Gallwey hirsutism score; NA: Data not Available; HOMA-IR, homeostasis model assessment of insulin resistance; HOMA-%B, homeostasis model assessment of beta-cell function (insulin secretion).

### Genotyping and haplotype determination

Discovery genotyping was performed on three *DKK1* single nucleotide polymorphisms (SNPs) (rs2241529, rs1569198, rs2288335) and four *DNAJB1* SNPs (rs7003, rs1803768, rs4926222, rs3962158) selected using genotype data of the CEU (Utah residents with ancestry from northern and western Europe) population of the HapMap database (release 24, http://hapmap.ncbi.nlm.nih.gov/). These SNPs were selected because they are predicted to tag SNPs across the entirety of each gene, plus 10 kb upstream and 10 kb downstream. These SNPs capture 21 of 26 (81%) of the CEU HapMap SNPs at r^2^>0.8 for the two genes. Replication genotyping was performed on two of the discovery SNPs; rs1569198 from *DKK1* and rs3962158 from *DNAJB1*. All genotyping was performed using Applied Biosystems Taqman Assays-on-Demand (Applied Biosystems, Foster City, CA) according to manufacturer's instructions.

Haploview (version 4.1) was used to calculate linkage disequilibrium (LD, the D' statistic) between each pair of SNPs and determine haplotypes and their frequencies [15]. The solid spine of LD algorithm in Haploview was used to determine haplotype blocks. Only subjects whose haplotype assignment was >95% certain were analyzed.

### Statistical analysis

Unpaired t-tests and chi-square tests were used to compare clinical characteristics between cases and controls; quantitative traits were log- or square-root-transformed as appropriate to reduce non-normality. Data are presented as median (interquartile range).

Genotypic association with PCOS status was evaluated using logistic regression, adjusting for recruitment site, BMI and age. Association between genotype and quantitative traits (conducted separately in cases and controls) was performed using linear regression adjusting for site, age and BMI in all analyses except those in which BMI was the dependent variable, wherein analyses were adjusted for site and age. To handle multiple testing in the discovery cohort significance was taken as *P*<0.008, considering that we analyzed two LD groups of SNPs (one per gene) against three families of traits (PCOS diagnosis, androgens and metabolic traits), yielding a Bonferroni correction factor of 6 (i.e. six independent comparisons). In the replication cohort, significance was taken as *P*<0.05, because the goal was to confirm the associations made in the discovery cohort. To limit multiple testing in the replication cohort, we only examined SNPs that displayed associations in the discovery cohort.

## Results

### Discovery cohort

**Figure 1:**
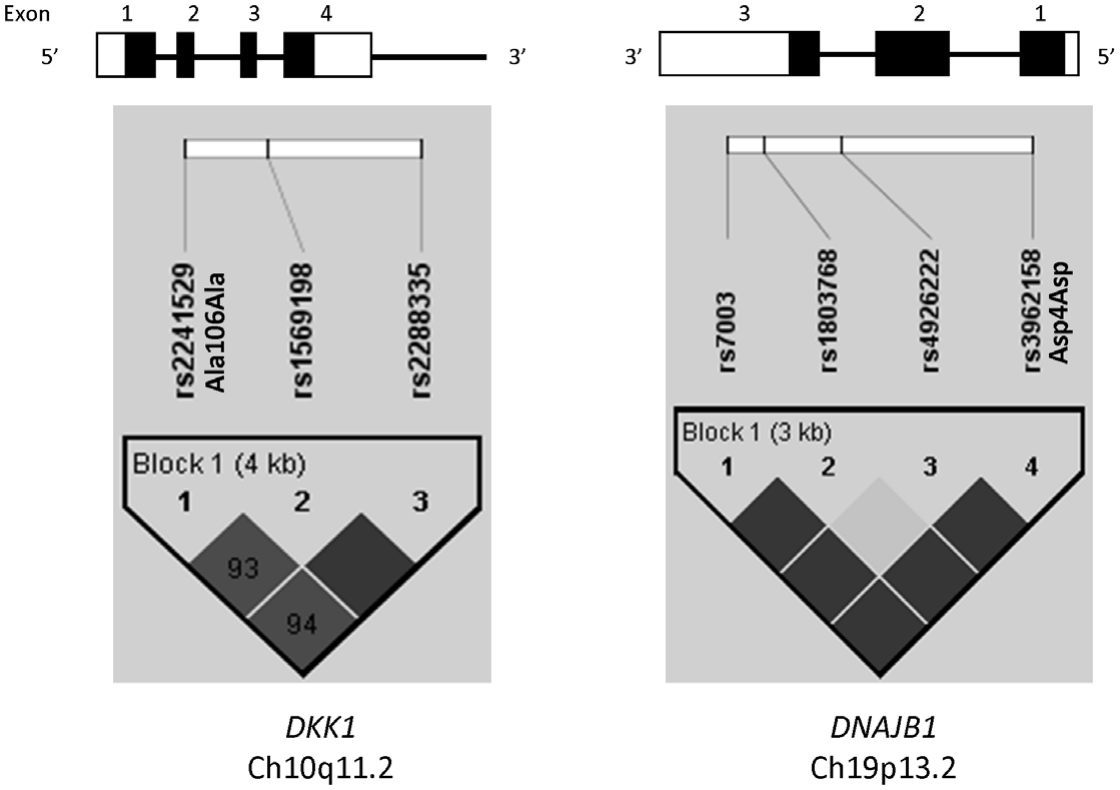
Gene structure and linkage disequilibrium plot for *DKK1* and *DNAJB1*. The gene structure is shown at top, with exons shown as filled boxes, untranslated regions as unfilled boxes and introns as connecting lines. The locations of the genotyped SNPs relative to the exons are indicated. The linkage disequilibrium (LD) plot at the bottom displays D' values (%) for each pair of SNPs in the box at the intersection of the diagonals from each SNP. The solid blocks indicate D' = 1 for the corresponding pair of variants. The darker solid blocks indicate a logarithm of the odds (LOD) score ≥2 for the corresponding pair of variants; lighter solid blocks indicate a LOD score <2. Within each gene, SNPs were considered together in one haplotype block as indicated.

We genotyped three SNPs from *DKK1* and four SNPs from *DNAJB1* in the discovery cohort (Table 2), where the overall genotyping success rate was 95.4% (ranging from 95.1–97.0% for individual SNPs). LD among markers (D') in DKK1 ranged from 0.93 to 1.0 (average pairwise D' of 0.96), while LD in *DNAJB1* ranged from 0.03 to 1.0 (average pairwise D' of 0.54). Haploview generated a single haplotype block including all three markers in *DKK1* and a single block encompassing all four markers in *DNAJB1* (Figure 1).

**Table 2:** SNP and haplotype information for the *DKK1* and *DNAJB1* gene regions in the discovery cohort.

Variant	Location	Alleles	PCOSMAF	Control MAF	Overall MAF
***SNPs***					
***DKK1***					
rs2241529	Exon 2 (Ala106Ala)	G/A	0.41	0.46	0.43
rs1569198	Intron 3	A/G	0.49	0.47	0.48
rs2288335	3′ region	G/A	0.08	0.09	0.08
***DNAJB1***					
rs7003	3′ UTR	G/A	0.12	0.15	0.13
rs1803768	3′ UTR	G/A	0.04	0.06	0.05
rs4926222	Intron 2	G/A	0.16	0.15	0.16
rs3962158	Exon 1 (Asp4Asp)	G/A	0.31	0.28	0.30
***Haplotypes***					
***DKK1***					
1		G-G-G	0.48	0.45	0.47
2		A-A-G	0.41	0.43	0.41
3		G-A-A	0.08	0.08	0.08
***DNAJB1***					
1		G-G-G-G	0.41	0.42	0.41
2		G-G-G-A	0.31	0.28	0.3
3		G-G-A-G	0.16	0.16	0.16
4		A-A-G-G	0.08	0.09	0.08

The order of SNPs in the haplotypes corresponds to the order of SNPs in the table. MAF  =  minor allele frequency.

SNPs in *DKK1* and *DNAJB1* were not associated with PCOS status. In women with PCOS, one SNP in *DKK1* (rs1569198) was associated with total testosterone, with major *A* allele carriers having increased testosterone levels (*AA/AG*: 2.74 (1.21) vs. *GG*: 2.36 (0.90) nmol/l; P = 0.0035). PCOS women carriers of the minor *T* allele at rs3962158 (*Asp4Asp*) in exon 1 of *DNAJB1* had increased insulin (*CC*: 78.0 (92.4) vs. *CT/TT*: 108.0 (124.5) pmol/l; P = 0.0031); increased HOMA-IR (*CC*: 1.72 (1.96) vs. *CT/TT*: 2.34 (2.39); P = 0.006) and increased HOMA-%B (*CC*: 145.2 (99.2) vs. *CT/TT*: 177.2 (108.6); P = 0.004). These SNP associations were not observed in the control women. No associations with BMI were observed.

Three common haplotypes (frequency >5%) were identified for the *DKK1* gene region (Table 2). No *DKK1* haplotype was associated with PCOS status or quantitative traits at our selected level of significance (P<0.008). Four common haplotypes were identified for *DNAJB1* (Table 2). PCOS women carriers of haplotype 2 had increased insulin (non-carriers: 78.0 (92.4) vs. carriers: 108.0 (120.9) pmol/l; P = 0.006), HOMA-IR (non-carriers: 1.76 (1.90) vs. carriers: 2.33 (2.43); P = 0.003) and HOMA-%B (non-carriers: 146.3 (97.7) vs. carriers: 176.6 (113.7); P = 0.002). This haplotype carries the minor allele of rs3962158, which was also associated with each of these traits in the single marker analysis. These haplotype associations were not observed in the control women.

### Replication cohort

We selected the two significant SNPs from the discovery phase of the study for replication. The genotyping success rate for rs1569198 was 95.7% and for rs3962158 was 97.3%, with 100% concordance observed in duplicate samples run both within the replication study samples, and across the replication and discovery study samples. The minor allele frequencies of rs1569198 (PCOS 0.48, control 0.51, overall 0.49) and rs3962158 (PCOS 0.31, control 0.30, overall 0.31) were the same as the frequencies observed in the discovery cohort. We observed association (additive model) between *DKK1* SNP rs1569198 and dehydroepiandrosterone sulfate (DHEAS), with increasing copies of the *A* allele correlating with increasing DHEAS (*AA*: 2229.0 (1551.3), *AG*: 2099.0 (1373.8), *GG*: 1978.0 (1520.0) nmol/l; P = 0.05). We did not replicate the association between total testosterone and rs1569198. We did, however, replicate the significant association between carriers of the minor *T* allele at rs3962158 of *DNAJB1* with increased HOMA-IR under the same dominant model (*CC*: 2.57 (1.65) vs. *CT/TT*: 2.77 (1.98); P = 0.036).

## Discussion

In an attempt to circumnavigate arbitrary bias introduced in the selection of candidate genes for PCOS, we evaluated mRNA and protein expression data reported from several PCOS tissues in order to identify novel susceptibility genes, *DKK1* and *DNAJB1*. These results suggest that variation in gene expression may be an important factor in identifying relevant PCOS pathways. This strategy was employed to select the 17β-hydroxysteroid dehydrogenase type 6 (*HSD17B6*) gene, which exhibited increased activity in PCOS ovaries, as a candidate gene for PCOS [16]; we and others ultimately replicated association of variants in this gene with metabolic phenotypes of PCOS [17], [18]. Others have performed network analysis on PCOS microarray data to identify pathways that may be most relevant to the development of the disorder [19].

A *DKK1* variant, rs1569198, was associated with testosterone levels in PCOS subjects in our discovery cohort, with carriers of the major allele having an elevated testosterone level. This result is consistent with the observation that a murine homolog of DKK1 plays a role in testicular testosterone production [20]. *DKK1* is expressed in a number of tissues including ovary, testis and adipose tissue, with increased expression in PCOS ovarian theca [4]. *DKK1* has been identified as one of the most upregulated genes in testosterone responsive tissues, such as the dermal papilla [21], suggesting its expression may be regulated by androgens. In a recent report designed to identify proteins that act in androgen-dependent hair loss, *DKK1* was reported as a dihydrotestosterone-inducible transcript, and also caused apoptosis in follicular keratinocytes [21]. These data suggest a role for DKK1 in the regulation of cell cycle in androgen responsive tissues, and further studies in ovary, particularly ovarian theca may confirm this. In a study of genetic determinants of bone phenotypes in men, rs1569198 was associated with hip axis length [22], which indicate potential roles for *DKK1* and this SNP in particular in a number of pathways, including those under hormonal regulation.

That we did not replicate the association of rs1569198 with testosterone deserves comment. The replication of associations such as these presents several challenges; the most important is the accumulation of an appropriate replication cohort, which must consist of an equal or large sample size of ethnically matched subjects, with similar disease phenotype and quantitative trait measures. The appropriateness of our replication cohort is substantiated by our replication of the association between rs3962158 and HOMA-IR. The replication of genetic association with testosterone poses a particular challenge, given the difficulty in precisely measuring this trait, particularly in women [23], [24]. In the replication cohort, we identified a nominal association of the major allele of rs1569198 with increased DHEAS, representing either a coincidence or supporting a role in androgen production or clearance. If the SNP does affect both testosterone and DHEAS levels, the lack of observation of association with both traits in both cohorts may be related to statistical chance or imprecision of androgen measurement in women.

Of particular interest as a candidate identified via differential expression was *DNAJB1* as it lies in the previously identified PCOS linkage region (microsatellite marker D19S884) on chromosome 19p13.2 [10] and acts as a molecular chaperone. Androgen receptor function (including ligand binding and nuclear translocation) depends on its interaction with heat shock proteins and their co-chaperones [25]. *DNAJB1* acts as a co-chaperone in a number of pathways including androgen receptor and glucocorticoid receptor signaling. An androgenic protein co-chaperone, *DNAJB1* is under transcriptional regulation by insulin, with increased hepatic expression demonstrated under conditions of reduced insulin [9]. *DNAJB1* may thus represent a common factor at the nexus of both the androgenic and insulin pathways that are frequently dysfunctional in PCOS.

The functional role of the associated *DKK1* and *DNAJB1* SNPs is unknown. The *DKK1* SNP rs1569198 is an intronic SNP, and the *DNAJB1* SNP rs3962158 does not change the amino acid at position 4. These SNPs, purposefully selected as tagging SNPs, may not be causal, but may be in LD with functional variants elsewhere in their genes or in the promoters. In particular, HapMap data indicate that the *DKK1* SNP is in LD with several SNPs in the promoter region of the gene. Tissue specific gene expression studies in genotyped subjects should be undertaken to further elucidate the role of variation in these genes on gene expression.

Despite many candidate gene studies in PCOS, few biologically selected candidates have been replicated. The use of expression data from PCOS subjects may result in the identification of novel susceptibility loci. In the present study, we used published expression data to select two putative candidate genes for analysis, *DKK1* and *DNAJB1*. By using a discovery and replication approach we have identified and replicated *DNAJB1* as a potential gene important in the insulin resistance of PCOS. While not associated with PCOS itself, genetic variation in *DNAJB1* and perhaps *DKK1* may act as modifiers, affecting the metabolic and androgenic pathways of PCOS, respectively. In conclusion, gene expression data appears to be a useful source of candidate genes in complex and poorly understood diseases such as PCOS.
